# Vegetarian Diets, Ayurveda, and the Case for an Integrative Nutrition Science

**DOI:** 10.3390/medicina57090858

**Published:** 2021-08-24

**Authors:** Archana Purushotham, Alex Hankey

**Affiliations:** 1 Department of Neurology, Baylor College of Medicine & Michael E. DeBakey VA Medical Center, Houston, TX 77030, USA; 2 School of Biology, Faculty of Science, MIT World Peace University, Pune 411038, Maharashtra, India; alexhankey@gmail.com

**Keywords:** stroke, diet, vegetarian, vegan, Ayurveda, dosha, prakriti, integrative nutrition

## Abstract

Two recent studies of the health effects of vegetarian diets reported conflicting results: the EPIC-Oxford study reported a significant increase in strokes among vegetarians compared to meat-eaters among a predominantly Caucasian cohort, while another, performed on Taiwanese Buddhists, reported significantly lower incidence of strokes among vegetarians. This was doubly puzzling given the pronounced decrease in cardiovascular events among the EPIC-Oxford group. In this article, we make a detailed comparison of the actual dietary intake of various food groups by the cohorts in these studies. We then use the nutritional principles of Ayurveda—traditional Indian medicine—to show how these apparently contradictory results may be explained. Systems of traditional medicine such as Ayurveda possess profound knowledge of the effects of food on physiology. Ayurveda takes into account not just the type of food, but also multiple other factors such as taste, temperature, and time of consumption. Traditional cuisines have evolved hand in hand with such systems of medicine to optimize nutrition in the context of local climate and food availability. Harnessing the experiential wisdom of these traditional systems to create an integrative nutrition science would help fight the ongoing epidemic of chronic lifestyle diseases, and improve health and wellness.

## 1. Introduction

Vegetarianism and veganism are diets growing rapidly in popularity not only because of perceived health benefits, but also because of social justice and sustainability concerns [1,2]. The EPIC-Oxford study was a longitudinal cohort study in the United Kingdom that examined the effects of diet—specifically a vegetarian diet—on cardio- and cerebro-vascular disease. Of a total of 48,188 enrollees, 16,254 were vegetarian. Over 18 years of follow-up, vegetarians had a 22% lower rate of ischemic heart disease compared to meat-eaters. This was in line with prior research: the most consistent benefits of vegetarianism have always been in cardiovascular health [3,4].

Cardiovascular and cerebrovascular disease are closely linked. They share a common pathophysiology, and have identical risk factors and principles of prevention and treatment. It was therefore rather surprising when the EPIC-Oxford study found a 20% increased risk of stroke, driven primarily by an increase in hemorrhagic stroke, in vegetarians [5]. The large size of the cohort and duration of longitudinal follow-up made this very unlikely to be an artefact.

Shortly afterward, another cohort study, this time from Taiwan, also reported on the influence of diet on stroke incidence. In two separate cohorts, one consisting of 1424/5050 vegetarians with 30,797 person-years of follow-up and another of 2719/8302 vegetarians with 76,797 person-years of follow-up, the authors found that stroke incidence was significantly lower among vegetarians. This was true in each cohort taken separately—i.e., the result was reproducible across the two cohorts. The reduced incidence included both ischemic and hemorrhagic strokes [6].

How do we reconcile these contradictory results from large, well-planned, and well-executed studies? Lower levels of low-density lipoprotein (LDL) and lower levels of vitamin B12 were both hypothesized as possible explanations for the greater incidence of strokes in the EPIC-Oxford study [2,5]. The authors of the second study actually showed that levels of LDL and vit. B12 were significantly lower in vegetarians; yet stroke incidence was lower. In fact, it was specifically in the subgroup with inadequate vit. B12 intake that there was a significant association between vegetarian diet and lower overall stroke [6].

In this Perspective, we take a deeper look at the reported diets of the study cohorts and adopt the perspective of Ayurveda, India’s traditional medical system, to try to explain these seemingly irreconcilable results.

## 2. Comparison of Cohort Diets

**Figure 1:**
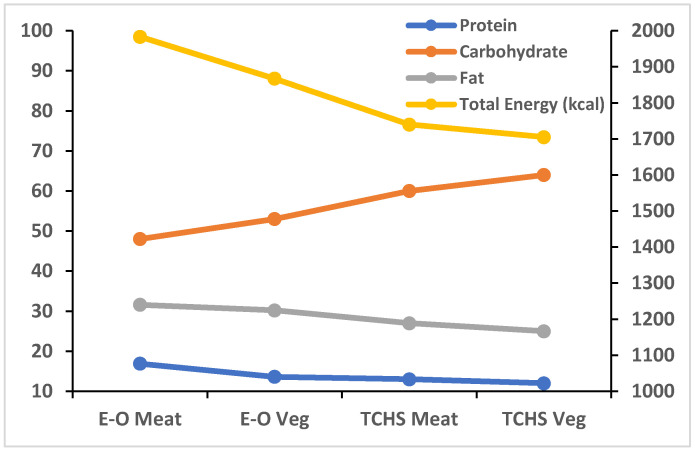
Comparison of the nutrient intakes in different diet groups of the EPIC-Oxford (E-O) and cohort 1 of the Taiwanese (TCHS) studies X-axis: Diet group; Y-axis (right): Total energy intake in kcal; Y-axis (left): Nutrient intake as percentage of total energy intake; Meat = Meat-eater; Veg = Vegetarian.

Figure 1 provides a comparison of the average nutrient intakes reported by the different diet groups in each study. Vegetarians in each study consumed fewer total calories, and a greater percentage of these was consumed as carbohydrates compared to meat-eaters. On the flip side, proteins and fats made up smaller percentages of the total caloric intake for vegetarians. Notwithstanding these commonalities, from the graph, it becomes apparent that there are fundamental differences in the patterns of nutrient intake between EPIC-Oxford’s predominantly European Caucasian subjects and the Taiwanese subjects of the second study (TCHS). The latter’s overall energy intake and proportion of protein and fat intake are lower, and that of carbohydrate higher, than the Oxford-EPIC subjects—so much so that the meat-eaters of TCHS consumed fewer calories, less protein and fat, and more carbohydrate than even the EPIC-Oxford vegetarians.

When the intakes of specific food groups are examined, the most striking difference between the two studies is in the consumption of soya products and legumes. TCHS vegetarians consumed 1.21 times as much plant protein, and 1.5 times as much soya-based food as TCHS meat-eaters. On the other hand, EPIC-Oxford vegetarians consumed 3.7 times as many legumes and soya-based foods as their meat-eating counterparts. In view of the aforementioned cultural propensity to eat a protein-heavy diet, this can be understood as an attempt to substitute for the missing animal protein with plant protein.

While such a simple substitution of one kind of protein for another may seem innocuous and even necessary from the perspective of Western nutrition science, Ayurveda, the system of traditional Indian medicine, believes that these foods have different effects on the body.

## 3. The Ayurvedic Perspective

Ayurveda is an ancient system of medicine that continues to be widely used in the Indian subcontinent today, especially for the treatment of chronic diseases such as stroke [7,8]. It uses dietary intervention as a cornerstone of therapy, both to maintain wellness and treat disease [9]. Additionally, because of the Indian subcontinent’s long and popular tradition of vegetarianism stemming from the principle of *ahimsa* or non-violence, the system possesses a good deal of collective experience in maintaining the health of vegetarians. Ayurveda extensively describes the effects of different foods on the body’s physiology, including meats and other non-vegetarian foods which it actively recommends in certain conditions. Looking at the dietary intakes reported above from an Ayurvedic perspective is therefore informative.

Ayurveda uses a broad, effectively systems-based three-way classification of physiological functions, called *doshas*: *vata dosha*, concerned with movement and input–output functions; *pitta dosha* concerned with turnover, i.e., digestion and metabolism; and *kapha dosha*, concerned with energy storage, growth, and lubrication [10]. Health depends on keeping *doshas* in balance [11]. Conversely, imbalances in *dosha* functions lead to disease; the pathogenesis of each disease condition is attributed to one or more *doshas* becoming progressively deranged [7,12].

In this context, each food may suppress or boost functions belonging to one or more *doshas*, thereby influencing important aspects of the overall physiology. This leads to increased or decreased susceptibility to specific medical disorders. Such effects of foods are mostly independent of the calorie–protein–carbohydrate–fat classification used in modern bioscience. Thus, while some protein-rich foods may boost a particular *dosha*, others may boost another *dosha*, or suppress the first, etc. [9]. Climate also plays a central role, since variations like windy/calm, hot/cold, and wet/dry can have deleterious effects on the *doshas*.

Revisiting the food intake of the EPIC-Oxford and TCHS dietary cohorts, the foremost difference between vegetarian and meat-inclusive diets is, of course, the absence of meat from the former. Meats overall tend to increase *kapha dosha*. Therefore, a meat-less diet, compared to a meat-eating diet, would in the long run decrease the occurrence of *kapha* disorders, which include metabolic syndrome disorders such as hypertension, diabetes, hyperlipidemia, and ischemic heart disease [13]. As previously noted, this is borne out by several research studies [3,14].

Now let us consider the effects of the much greater legume and soya-based food intake seen among the EPIC-Oxford vegetarians. In general, legumes including soya, chickpeas, and kidney beans greatly increase *vata dosha*. Indian (and other Eastern) vegetarian diets do contain quantities of legumes, but favor the less *vata*-genic ones such as the mung, highly proteinaceous urad, toor, and masoor beans. These are also prepared with special care: their effects on *doshas* are typically balanced out by liberal use of contrarily acting spices, and eating them hot and very well cooked [15]. Not adequately tempered, chronic *vata*-boosting can lead to diseases such as stroke and other neurological disorders known in Ayurveda as *Vata vyadhis* (“The *Vata* Diseases”) [16,17,18].

Fermentation and some of the other processing techniques used to prepare soya products, such as soy sauce, tempeh, and soy meats, can lead to boosting of *pitta dosha*. A combined aggravation of *vata* and *pitta* is far more potent than that of either alone, especially when *pitta* is said to obstruct *vata*, and may in particular predispose to hemorrhagic strokes [19,20]. The increased stroke incidence observed in the EPIC-Oxford vegetarians, but not the TCHS vegetarians, is thus not surprising when viewed through the lens of Ayurveda.

## 4. Vegetarianism and Ayurveda

Traditional cuisines have evolved over millennia, informed by local medicinal knowledge to optimize health and well-being in the context of local climates and value systems. Traditional Indian cuisines, notwithstanding the wide variety across the subcontinent, are based on Ayurvedic principles, and have a common underlying theme of combining food types to promote balance of *dosha* functions in the context of local climate, season, and availability. These take into account not just the type of food, but innumerable other factors—including taste, texture, temperature, and time of consumption—that contribute to the effects of foods. For example, the *Kshemakutuhalam*, a 16th century Ayurvedic text on dietetics, describes traditional Indian cuisine, including combinations of ingredients to be used in different recipes and their health benefits, at length [21]. The three authoritative texts of Ayurveda each recommend seasonal modifications to the diet. The *Ashtanga hridayam*, one of the three, has an entire chapter devoted to *Ritucharya*—i.e., seasonal lifestyle including diet [22].

Another important Ayurvedic consideration for a meat-eater planning to turn vegetarian is the concept of *Satmya*—the importance of habituation [23]. Any change in diet from one that an individual was previously accustomed to, should be introduced gradually, in stages [24]. An abrupt change to a new diet—even if it is healthier than the prior one—can lead to ill health (“*Asatmyajanya roga*”) [23]. Hence, Ayurveda would recommend that the transition to vegetarianism be a gradual one [24].

Vegetarians who do not come from a culture with a long vegetarian tradition, looking to develop their own healthy meal plans, may benefit from considering such nutritional perspectives of traditionally vegetarian cuisines.

## 5. Conclusions

Systems of traditional medicine such as Ayurveda possess rich knowledge of the effects of various foods on physiology, accumulated over centuries of keen observation. As evidenced above, Ayurvedic principles can shed entirely new light on an otherwise inexplicable clinical observation or outcome.

Ayurveda is a promising weapon against chronic disease [7,25,26]. Its extensive experience with holistic nutrition, together with that of other traditional systems of medicine, should be harnessed to create an integrative nutrition science to promote health and well-being.

## Data Availability

Data was only contained in the stated references.
